# Local Anæsthesia

**Published:** 1866-08

**Authors:** William R. Whitehead


### Local Anæsthesia.

BY WILLIAM R. WHITEHEAD, M. D.

The attempt to substitute local for general anæsthesia in extensive surgical operations, offers the attraction of novelty, and the inviting advantage of avoiding the occasionally fatal accidents resulting from the inhalation of chloroform. The attention recently accorded to the efforts of Richardson, of London, to introduce to the favorable notice of the profession local anæsthesia by means of ether spray, will sanction a review of the relative merits of local anæsthetics.

From the most remote periods attempts have been made to produce local insensibility to the pain of surgical operations. Narcotics were topically employed by the ancients with discouraging results. During the last century compression was greatly extolled; but the constriction of a limb by tight cords to lessen the pain of an amputation was as inefficient as it was inconceivably barbarous. Arnott, in England, rendered temporarily popular a freezing mixture of salt and ice. I have seen this application made by Velpeau, for the avulsion of toe-nails; and the pain which it occasioned was intense. The cold produced by the rapid evaporation of ether induced Guérard, about 1854, to cause to be constructed by Mathieu,* an instrument provided with a small syringe, which should project ether in a continuously fine jet upon any part where it was desired to lessen or abolish sensibility. Hardy, of Dublin, in 1853, recommended the evaporation of chloroform as a local anæsthetic.

Follin and Leconte, several years ago (Bulletins de la Societé de Chirurgie,) instituted a series of experiments to produce local anæsthesia.

By projecting a jet of ether on the skin, they produced insensibility of the part in less than a minute; and froze water in a small glass tube in less than two minutes. Great pain attended the application of the freezing mixture of salt and ice; and they further established that the complete anæsthetic effects which they obtained were in both cases due entirely to the congelation of the tissues. The success of these experiments caused ether sometimes to be used locally for minor operations, when the incisions were few, and involved only the integument. But the application of ether topically has unquestionably been greatly facilitated by the use of spray instruments; and if Richardson did not, a few years ago, meet with encouraging success in his endeavor to produce local anæsthesia by electricity, his adaptation of a nebulizer to the use of ether, though only an improvement on the method of Guérard, is productive of results that may retain this means of local anæsthesia among the recognized remedial agents which are exceptionally useful. In some of the recorded cases of success, as in the ovariotomy case of Spencer Wells, it was found necessary to administer chloroform to complete the operation; and furthermore, the experience of surgeons has not been confirmatory of the high encomiums lavished on ether spray in surgical operations of magnitude. I have recently used it in a plastic operation on a gentleman of this city, who had the choice of ether by inhalation or applied locally; chemically pure ether was used and applied by skillful hands. As the operation was performed by a few rapid incisions, and my object was only to materially diminish the sensibility, congelation of the tissues was not produced.

---

The impossibility of performing difficult dissections among frozen tissues must necessarily confine within restricted limits the indications for this anæsthetic method; and it will be difficult to cause surgeons to abandon the positive benefits of chloroform for the doubtful advantages of local anæsthesia by ether spray. Since the effect is due entirely to the cold produced, I would prefer rhigolene to ether; but reserve it only for minor operations. There is a great variety of spray-producing instruments, and the perplexity in the choice of a suitable instrument can only result from a failure to make simplicity the requisite quality in the selection. Richardson's or Clark's will answer.

In almost any drug-store can be found a little instrument called the "scent distributor," for many years familiarly known to the public, and used for perfuming apartments. This little instrument is the simplest variety of nebulizer, and consists of two glass tubes arranged at right angles to each other. If the smaller tube should be passed through a cork stopper into a small bottle containing ether, and the other glass tube attached to a rubber-pipe connected with two hollow rubber balls at short distances from each other, one used for compressing air, and the other, which is nearest the bottle, as an air-reservoir, we shall have a representation of Bergson's apparatus modified by Clark.—*Medical Record.*

